# Globally Estimated UVB Exposure Times Required to Maintain Sufficiency in Vitamin D Levels

**DOI:** 10.3390/nu16101489

**Published:** 2024-05-15

**Authors:** Richard C. Kift, Ann R. Webb

**Affiliations:** Department of Earth and Environmental Sciences, University of Manchester, Manchester M13 9PL, UK; richard.kift@manchester.ac.uk

**Keywords:** cutaneous synthesis of vitamin D, ultraviolet radiation, vitamin D irradiance climatology

## Abstract

A paucity of vitamin D is a common deficiency globally, with implications for many aspects of health besides the well-known impact on musculoskeletal health. The two sources of vitamin D are through oral intake, or through endogenous synthesis in the skin when exposed to ultraviolet radiation in sunlight. Assessing nutritional needs, whether by food, food fortification or supplementation, is aided by an understanding of local potential for cutaneous synthesis of the vitamin, dependent on latitude and climate, personal skin type and local culture. To aid these discussions we provide indicative exposure times for the maintenance of vitamin D status as a function of latitude, month and skin type, for the clear-sky case and all-sky conditions, for an ambulatory person wearing modest skirt/shorts and T-shirt. At latitudes greater than ±40 degrees, lack of available sunlight limits vitamin D synthesis in some months for all, while at the equator exposure times range from 3 to 15 min at noontime, for white and black skin, respectively. Rather than a sun exposure prescription, the data are intended to show where nutritional vitamin D intake is necessary, advisable, or can be mitigated by sun exposure, and allows for such advice to be personalized to account for different sub-groups in a multicultural population.

## 1. Introduction

It is established that vitamin D plays an essential part in maintaining a healthy musculoskeletal system; its anti-inflammatory, antioxidant and neuroprotective properties are beneficial to general health and there is increasing evidence it plays a role in a range of other diseases such as multiple sclerosis, as well as supporting the immune system [1,2,3,4,5]. The body has two sources of vitamin D: by ingestion through diet or supplementation, or by cutaneous synthesis on the exposure of unprotected skin to the UVB radiation in sunlight [6]. As modern diets are generally poor sources of vitamin D, this means for the majority of people that the main source is cutaneous synthesis. However, evidence shows that large proportions of the population have low vitamin D status some or all of the time [7,8]. Given the often-stated assessment that 90% of the body’s vitamin D supply is synthesized within the skin, low vitamin D status as determined by circulating 25-hydroxyvitamin D (25OHD) levels also implies a significant lack of exposure to the UV in sunlight. Yet encouraging more sun exposure contradicts long-running health campaigns, at least in white-skinned populations. This means that many national public health bodies have made the decision to fortify certain foodstuffs [9] or to recommend vitamin D supplements for some or all of the population [10,11,12]. Nonetheless, for many populations, the availability of significant UVB as well as the lack of local health services and availability of vitamin supplements would suggest that advice is needed to determine how to safely obtain/maintain vitamin D sufficiency using ‘little and often’ sun exposure in such a way as to avoid the health risks of sunburn and skin cancer. The data required to provide such a public health message are complex, especially as ‘little and often’ requires a personal prescription dependent on an individual’s characteristics and location.

The topic is further complicated by different definitions of vitamin D deficiency, defined by the levels of circulating 25OHD. Examples range from 25 nmol/L [10] to 75 nmol/L [11,12,13], while there is an increasing convergence on using 50 nmol/L as the boundary between deficient or insufficient vitamin D status, and an adequate or sufficient level [14]. The current study considers only maintenance of vitamin D status, that is, how to achieve a zero change in circulating 25OHD. The method of initially reaching a desired level is not addressed, nor is the precise target 25OHD, although it should avoid extremes of 25OHD (high or low) as these were not represented in the underlying clinical studies on which the methodology is based.

The link between UV exposure and circulating 25OHD is provided by a previously reported dose–response relationship [15] based on a systematic review of studies using artificial sources of UV radiation, and predominantly healthy, white-skinned participants; verification by a small number of sunlight studies is also provided. Application of this dose–response relationship to inform nutritional guidance depends on a range of factors that are both environmental and personal. The major environmental control is the availability of solar UVB radiation, itself determined by latitude, time of year (date) and weather, while the main personal factor is skin pigmentation i.e., skin type (white-, brown-, black-skinned) as defined using the Fitzpatrick system [16]. At the intersection of the environmental and personal are factors that affect how much available UV reaches the skin e.g., clothing that can be determined by temperature or culture; and the immediate surroundings e.g., city canyon or open field; time when exposure is taken and for how long. Our aim is to provide input to national vitamin D nutrition guidelines by illustrating the potential for maintaining vitamin D status through sun exposure as a function of latitude, time of year and skin type. As we show, this will allow a more nuanced and targeted approach to considerations of national food fortification, national supplementation policies, and sun exposure guidance, based on solar UV availability and tempered by the characteristics and cultures of the local population.

## 2. Materials and Methods

The initial global coverage of surface ultraviolet (UV) irradiance data were obtained from the Global Ozone Monitoring Experiment (GOME), quantified as the UV index (UVI), a measure of the erythema effective radiation. This was subsequently converted to the more appropriate Vitamin D effective UV irradiance. The data come from a series of instruments mounted on several ESA satellites from ERS-2 to Metop, providing a continuous data series from 2002 to the present, provided via the TEMIS portal (https://www.temis.nl/index.php, accessed on 1 September 2021). The satellite products used were the globally available clear-sky UVI, and the all-sky data for the regions where cloud data could be incorporated into the product. Note the UVI is not measured directly but is a product based on satellite measurements of ozone, with corrections applied for aerosols, solar distance, surface elevation and surface albedo (https://www.temis.nl/uvradiation/product/, accessed on 20 March 2024), which are used in a radiative transfer model to determine UV at the surface [17]. Hence a clear-sky, or maximum, value is available globally for every day of the year, and for this study, the UV was obtained using the climatological data from 2004 to 2020. The use of climatological data means that our results are based on general expectations and not exact UV for a particular day. This is especially so when cloud is included, but even for the clear-sky model, ozone varies across the years and from day to day within a month. Furthermore, aerosol loading was chosen to best represent the most populated regions of the world, but remains an overall estimate and does not capture the full impact of extremes in aerosol loading.

The satellite midday UVI values were first converted into units of standard erythema dose (SED), where 1 SED = 100 Jm^−2^ of erythemally weighted UV. For use in the equation derived in [15], this was converted to units of standard vitamin D dose (SDD), where 1 SDD = 100 Jm^−2^ of vitamin D weighted irradiance [18]. The SED/SDD ratio is not a constant and changes in line with variations in the solar spectrum, which are a function of SZA and column ozone. For this work, the SED/SDD ratio was determined using the Smarts 2.95 model [19] over a range of SZAs (0–90 degrees) and ozone values (200–450 DUs), showing that for UVI ≥ 2, the SED/SDD ≈ 0.5 [20]. When UVI < 2, the sun is low in the sky and any practical vitamin D synthesis is negligible. The ratio SED/SDD was therefore assumed constant and, for times and places where the average clear-sky midday UVI < 2 throughout a month, we defined a Vitamin D Winter (VDW)—that is practical sun exposure would be ineffective for vitamin D synthesis. The midday UVI data have been used as it is an easily defined time, typically represents the maximum UVI for the day (and hence minimum exposure time), and is a period when most people have a lunch break and so the potential to be outside for a short period.

To estimate the change in vitamin D status we used Equation (1) from [15] where change in 25OHD is dependent on exposure to UV in terms of SDD:

(1) $$\Delta 25{\mathrm{OHD}\;(\mathrm{nmolL}}^{-1})=9.51\ln\left(\mathrm{SDD}\right)-7.6\;$$

Equation (1) is not valid for single exposures and is best applied for periods similar to those on which the underlying studies were based. A period of one month has been used here. The equation is also strictly appropriate for ‘full body’ exposure (i.e., about 90% skin area) and for white skin types. Therefore, in the first instance we considered the simplest case of someone lying out unclothed in the sun at noon each day under a cloud-free sky and calculated the dose in SDD required to maintain vitamin D levels (Δ25OHD=0). The time to acquire this dose was then determined by the local UV irradiance.

From (1) we obtain an average maintenance dose of ~2.33 SDD per month, that is, the total dose for which there is no change in 25(OH)D. This equates to a dose per day (DD) of (2.33/days in month). A 10 min dose (D_10_) was then calculated as a function of day and location from TEMIS data, and averaged over each month. In a clear-sky situation, the UVI is essentially constant at solar noon, and can be taken as such for this brief 10 min period. D_10_ was used to determine the maintenance exposure time required at noon, noting that when lying horizontal, only half the surface skin area can be exposed at one time, so to give all skin the same dose effectively doubles total exposure time.

The noon exposure time (D_t_) in (minutes) for any location is given by:

(2) $$D_{t}=2\times 10\left(\frac{D_{D}}{D_{10}}\right)$$

This simple calculation, replicating the underlying studies from the dose–response curve, was then scaled in three consecutive ways to make it more realistic and applicable to everyday life. First the exposure time was adjusted to represent random vertical orientation of the skin surface, i.e., someone standing up and walking around rather than lying flat on the ground. This was achieved using a horizontal to vertical scaling employed previously [21,22], and that is itself a function of SZA. Skin surface area was then assumed to be 35% (equivalent to modest shorts/skirt and T-shirt), since this both represents further underlying clinical studies [23,24], and has been shown to enable vitamin D needs to be met without the risk of sunburn [25]. For skin area, a simple linear scaling factor was used as there is insufficient information regarding potential differences in vitamin D production as a function of body region to warrant anything more complex, although there is some evidence that pre-exposure and tanning are the main factors affecting rates of production in skin [26,27]. To adjust for skin type, data were taken from previous studies of white Caucasian (skin types I–IV) and skin type V individuals [24,27]. Equivalent data were not available for skin type VI, so these times were adjusted based on time for a minimum erythema dose (MED) for the Fitzpatrick skin type [16]. Exposure for skin type V was scaled by a factor of 2.5 compared to white Caucasian skin types, while for skin type VI a factor of 4 was used.

When adjusting for darker skin types at higher latitudes, there was potential for exposure times to become long enough to challenge the assumption of constant irradiance during the exposure. Corrections to account for this were calculated as a function of latitude and season, and, although small, were applied where appropriate; as an example, for an hour exposure time in mid-latitudes in spring, the correction is a factor of 1.03.

One further factor that needs to be considered is the effect of clouds which on average reduce the UV levels and hence increase the time needed to obtain a maintenance dose. To examine this, the daily mean cloud cover data were used to estimate the reduction in available UV and the consequent increase in maintenance dose times.

## 3. Results

**Figure 1:**
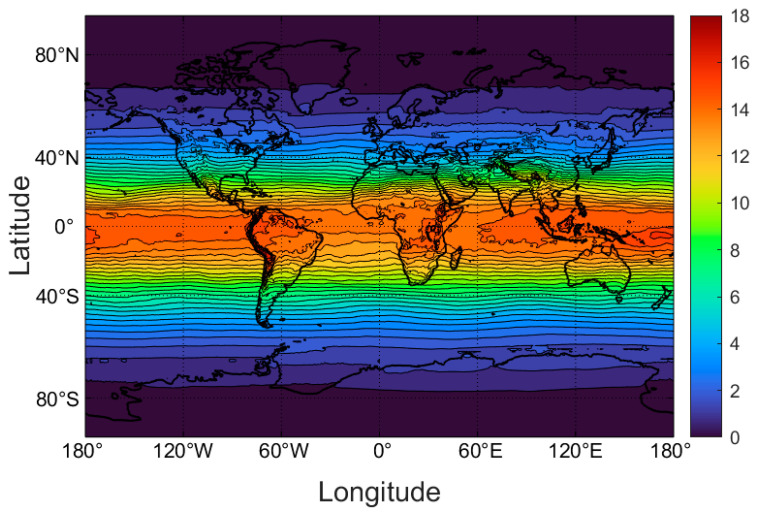
Midday mean clear-sky UVI values averaged over the days of March, 2004–2020. While generally latitude-dependent, the UVI is also influenced by column ozone (less in the Southern Hemisphere, especially at high latitudes), and altitude (most noticeable with the Andes on the west coast of South America).

The original UVI dataset from TEMIS consists of a global grid (720 × 1440) of values with 0.25-degree steps in both latitude and longitude. This is shown in Figure 1 for the month of March.

**Figure 2:**
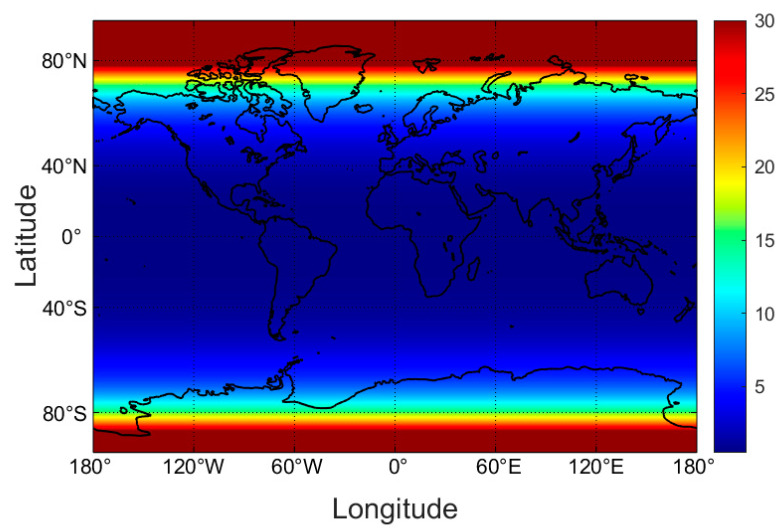
March maintenance dose times (mins) for clear-sky conditions at noon, white skin types, 35% skin area exposed, horizontal body, one side exposure time (note: to get the dose on front and back (=full body) requires turning over, which doubles the total time needed to gain exposure). Averaged over every 0.25 degrees of latitude.

**Figure 3:**
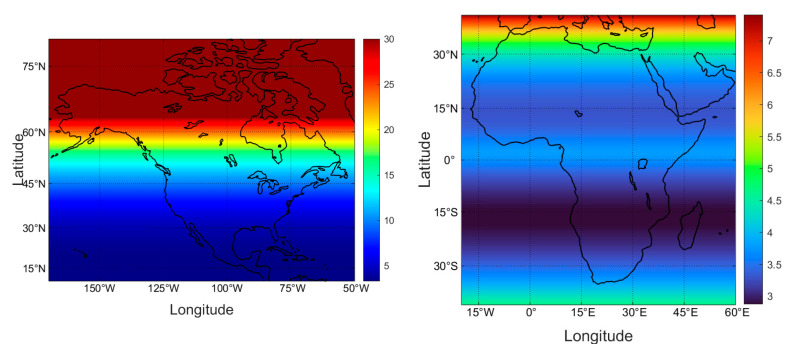
March maintenance dose times (mins) for clear-sky conditions at noon, white skin types, 35% skin area exposed, vertical body, average all sides, for Africa and North America. Note the different color scales. The high latitude brown region of North America indicates where the UVI is below the defined Vitamin D winter values.

The exposure times for a horizontal body (one side) with white skin and 35% skin surface area exposed (Figure 2) was calculated for each pixel for each month as described in the Methods, based on the UV climatology illustrated in Figure 1 for the month of March. The step of converting to an upright body and averaging exposure over all sides (assuming random motion) is illustrated in Figure 3 for two sub-regions of the globe, enabling a greater degree of resolution in the maintenance dose time. Other sub-regions are shown in the Supplementary data (Figures S1–S4). The data were then averaged at each 10 degrees of latitude to provide simple tables of indicative exposure times for the conditions stated in each case. Other than those presented in the main text, tables (horizontal exposure times for two sides, and vertical body exposure times, clear- and all-sky conditions, for each of skin types I–IV, V and VI) are provided in the Supplementary data (Tables S1–S8). Data in the tables are given in whole minutes since it is intended as a broad guide, and in a practical sense, the general population is unlikely to time their exposures to fractions of a minute.

### 3.1. Clear Sky

The maintenance dose exposure times for the month of March (equinox) are shown in Figure 3 for Africa and North America. The average exposure times at every 10 degrees of latitude across the globe, derived from these more detailed data, are shown in Table 1. Note that for white skin, the maintenance dose time ranges from 3 to 15 min (equator to high latitudes) in these ideal conditions, for locations and seasons that are not experiencing the vitamin D winter. The lack of symmetry at high latitudes in the southern hemisphere is a result of strong Antarctic ozone depletion occurring in the austral spring. At tropical latitudes, the sun is directly overhead at the equinoxes (March and September) and is furthest from the equator in June and December, providing for the double minimum exposure times.

The same pattern generally holds for skin type V (Table 2), but with the requirement for increased exposure times at all latitudes and in all seasons compared to skin types I–IV. This is expected since [16] showed that those with skin type V require approximately 2.5 times more UV exposure than those with skin type II to produce the same change in circulating 25OHD. This means that while maintenance of 25OHD in our standardized conditions (35% skin area, vertical body, noontime) remains possible, the times involved become more than ‘casual exposure’, exceeding 30 min at high latitudes in some or all months. Maintenance of vitamin D status through daily exposure of less than 15 min is only viable in the region ±30 degrees latitude.

In the case of skin type VI (Supplementary data, Table S1), the effect of higher latitudes and season is even more pronounced, with 15 min exposure times exceeded in some months at all latitudes outside ±10 degrees of latitude and 30 min exposure times exceeded in at least some months at latitudes 30 degrees and higher.

### 3.2. Cloudy Skies

**Figure 4:**
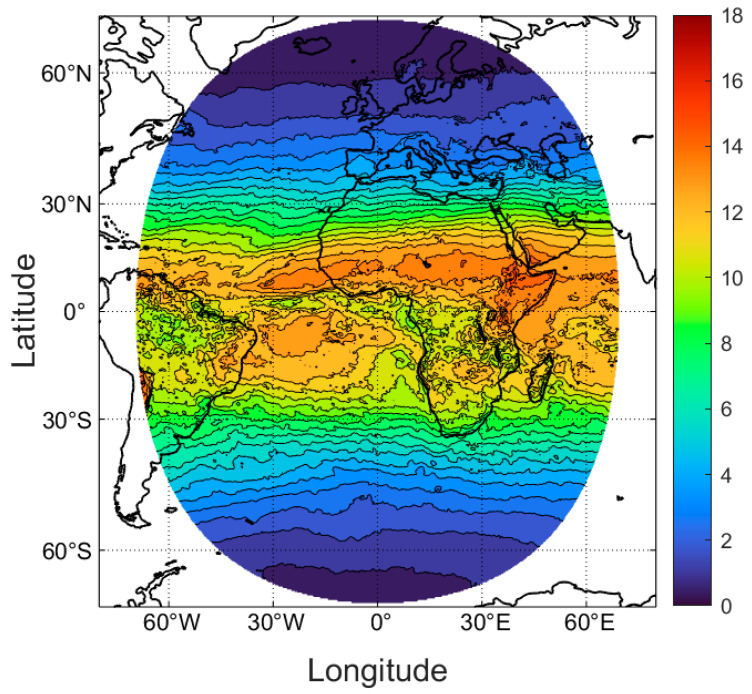
Midday mean all-sky UVI values averaged over the days of March, 2004–2020.

The idealized case of clear skies and a flat unobstructed horizon will result in the greatest UV irradiance and the shortest exposure times. In reality, cloud intermittently reduces UV irradiance, while obstructions such as buildings or trees can obscure either the sun, placing a body in shade, or parts of the sky that also contribute to overall exposure. This is particularly so at short UV wavelengths where much of the radiation is scattered or diffuse and is incident on a body from all around. Local obstructions cannot be accounted for in the model, but cloud has been included in the calculations in this section, using the TEMIS all-sky dataset for regions where it is available (Figure 4).

The exposure times based on all-sky climatology (Figure 4) for skin types I–IV are shown in Table 3. The latitudinal averages for the all-sky data are only calculated over the longitudes where all-sky data are available. At higher latitudes, this means the longitude range is very restricted, and even at the equator, the data only truly represent ~140 degrees of longitude. Given that the all-sky data are less zonally consistent than the clear-sky data, and the tables are based on limited data, these tables are indicative only with more uncertainty in the maintenance dose exposure times than for clear skies. This stems partly from the natural variability introduced by cloud, and partly from the limited all-sky data available. Comparing Table 3 with Table 1 shows that the addition of cloud does not have a consistent effect across the globe, with the impact of cloud being dependent on local climate, that is, the frequency of cloud-covered skies in different seasons, and to some extent the type or depth of cloud (https://www.temis.nl/uvradiation/product/clouds.html, accessed on 20 March 2024). The presence of cloud extends the zonally averaged maintenance exposure times by about 15% in equatorial regions, increasing to up to an additional 60% time at high latitudes. Nonetheless, for white skin types, a maintenance dose is still an achievable target, even at high latitudes at times outside the VDW. This is not the case for skin type VI (Table 4), for which a 15 min exposure time is required in equatorial regions under all-sky conditions, and this increases to well over an hour at higher latitudes.

## 4. Discussion

Globally, there are significant levels of vitamin D deficiency in many populations and age groups, contributing to a range of health problems. Whilst diet, food fortification and supplementation provide a potential oral solution, an understanding of the role that free sunlight can play in helping to reduce vitamin D deficiency is also part of the solution. The optimized balance between nutritional intake of vitamin D in all its forms, and cutaneous synthesis, will depend on location, local population characteristics, and the local economy and health care facilities. The results here facilitate that discussion by illustrating what is, and is not, possible in terms of maintaining 25(OH)D levels through solar induced vitamin D synthesis as a function of latitude and skin type.

A primary limitation on maintenance of vitamin D status through sun exposure is lack of solar radiation in the winter season. The VDW starts to become significant at latitudes around 40 degrees, and its duration increases with increasing latitude. With ample supply (e.g., in summer), vitamin D can be stored in the body [28] and is then released when supply is low (e.g., in winter), modulating the response of circulating 25OHD to seasonal supply that nevertheless results in the oft-observed seasonal cycle in 25OHD at higher latitudes [29,30]. Hence a lack of any possibility of a maintenance dose from solar exposure implies either that vitamin D status will begin to decline, or another source of the vitamin must be found, through diet or supplementation. One potential solution would be to increase exposure times (without sun burning), or increase skin area exposed, in the summer months to increase 25OHD enough to cover the following VDW decline. It has been shown that this is possible for the UK for those of skin types I–IV [24], but becomes more challenging for skin type V [25]. An alternative is to encourage the use of supplements at certain times of the year, or to increase the baseline dietary intake of the whole population throughout the year, a target that would imply food fortification.

Interacting with the underlying local climate is the personal characteristic of skin color. Skin type VI represents those with the most deeply pigmented skin, evolutionarily adapted for tropical sunlight. When moved to higher latitudes, maintaining vitamin D status through sun exposure becomes challenging, particularly with modern lifestyles that have replaced outdoor, agricultural occupations with indoor, industrial and service occupations. The latter issue of changing lifestyles applies to all skin types, but modern practices exacerbate the latitudinal effect most acutely for those with considerable constitutive pigmentation. Table 3 and Table 4, plus Supplementary Table S2 for skin type VI, show that maintenance of vitamin D with noontime sun exposure of less than 30 min a day (with 35% skin area exposed) cannot be achieved all year round at ±30 degrees latitude and higher for skin type VI, at ±40 degrees latitude and higher for skin type V, even in some months when sufficient sun is available, and at ±40 degrees latitude and higher for skin types I–IV due to the VDW. If sun exposure of around 15 min or less at noon is all that is achievable, then the latitude band is decreased by 10 degrees of latitude for each skin type except skin types I–IV, which remain at ±40 degrees. The ability to maintain vitamin D status by sun exposure becomes a complex interaction between location (latitude and climate), personal characteristics (skin type), both accounted for here, plus immediate surroundings and occupational and local culture, which are not included in the tables provided.

There are many caveats to the results presented here and we stress that they are intended as indicative guidelines for assessing nutrition requirements, and not as prescriptions for sun exposure. The tables provide data at every 10 degrees of latitude and, in the all-sky case over all weather conditions, effectively average cloudiness for the latitude. Using the all-sky exposure times means that, in some days, exposure may be less than expected (more or thicker cloud than average), and sometimes it will be more than expected (less or thinner cloud). Since the vitamin D maintenance dose for all skin types is very much sub-erythemal, occasionally gaining as much as 60% more UV (the extreme of using the all-sky dose time on a clear day) should not present an issue. The all-sky exposures will then average out over the period of a month. To account more precisely for latitude, one can extrapolate between the latitude bands in the tables.

Sun exposure can be gained at any time of day, although at close to sunrise and sunset at any time and place, when the sun is low in the sky, negligible vitamin D synthesis will be initiated (equivalent to the VDW situation). Away from the noon hour, the solar irradiance decreases, so exposures taken at other times of day would need to be longer than at noon. The tables presented here therefore indicate the minimum exposure times that would serve to maintain vitamin D status. Furthermore, all calculations assume an unobstructed horizon and no shading, which is often not the case, especially in a cityscape. Shading, or a significantly obstructed horizon, will both decrease exposure and increase exposure times.

The 35% skin area exposed, used throughout as a reference skin area, represents the exposed skin area used in the clinical studies that underpin some of this work [22,23]. However, the skin area exposed in most of the studies contributing to the dose–response curve was ‘whole body’ (effectively ~90%). Since this is not a practical suggestion for the general population in daily life, we have reverted to the 35% skin area and assumed a linear scaling of vitamin D synthesis with skin area. It is also recognized that some cultures and some occupations do not encourage even this degree of skin exposure, and the tables of sun exposure times may not be applicable. Where this is the case and skin exposure to sunlight is severely limited, then oral intake of vitamin D should be increased.

Further uncertainties that we have not addressed include other personal factors such as age and ethnicity (beyond skin color), and the complexities that come from prolonged skin exposure to solar radiation. The ability of aged skin to synthesize vitamin D has often been assumed to be compromised by a decline in the underlying precursor 7DHC with age [31]. More recent work with new analytical techniques has shown that the healthy, ambulatory aged have no detriment in skin synthesis of vitamin D [32], implying that our results are relevant throughout the life course. There may also be ethnic variations in the requirement for vitamin D, at least for bone health [33], though in this case the circulating 25OHD responds as expected, but the impact on bone mineral density differs between ethnicities. This would imply suitable selection of a target level of circulating 25OHD by ethnicity, but the sun exposure for maintaining that level (zero change in 25OHD) is still addressed by the current work. The dose of UV radiation required to maintain vitamin D status is small and the time short (a few minutes) if the dose is daily, a finding that concurs with previous work that has approached this question from a variety of directions (e.g., dose to attain a nutritional intake equivalent [34], or to prevent the nadir of the seasonal cycle being at deficiency level [35]). This avoids the complexities of vitamin D photochemistry in the skin that comes with extended exposure, as well as any temporary change in skin melanin content afforded by tanning [36]. If exposure has been extensive enough for these complicating factors to be relevant, then we consider it ample for maintenance of circulating 25OHD.

## 5. Conclusions

Vitamin D status, determined by circulating 25OHD, is the result of both oral intake of vitamin D and cutaneous synthesis in the skin following exposure to solar ultraviolet radiation. The balance between these two sources, and the sufficiency of their combined input, will depend on diet/supplementation, and on both availability of and exposure to solar UVR. The results presented here provide guidance on the duration of noontime sun exposure that would provide for maintenance of vitamin D status, as a function of latitude and skin type. They are intended as a guide to aid in determining nutritional requirements, and should be used with additional knowledge of the local population characteristics and culture.

## Figures and Tables

**Table 1:** Daily exposure times in minutes, mean for the latitude given, for maintenance of 25OHD levels under climatological clear-sky conditions, clear horizon, at noon. White skin (ST I–IV), 35% skin area exposed, vertical body, averaged over all sides exposed simultaneously. W = Vitamin D Winter.

Lat/Mth	Jan	Feb	Mar	Apr	May	Jun	Jul	Aug	Sep	Oct	Nov	Dec
90	W	W	W	W	W	W	W	W	W	W	W	W
80	W	W	W	W	W	14	14	W	W	W	W	W
70	W	W	W	W	11	9	9	13	W	W	W	W
60	W	W	W	12	8	6	6	8	14	W	W	W
50	W	W	12	8	6	5	5	6	8	15	W	W
40	W	11	7	5	4	4	4	4	5	8	15	W
30	8	6	4	4	4	4	4	4	4	5	7	9
20	5	4	3	4	5	4	4	4	3	4	5	5
10	3	3	3	4	4	3	4	4	4	3	3	4
0	3	3	4	3	3	3	3	3	4	3	3	3
−10	3	3	3	3	4	4	4	3	3	4	3	3
−20	4	3	3	4	5	6	6	4	4	4	4	4
−30	3	3	4	5	8	10	10	7	5	4	4	4
−40	3	3	5	8	15	W	W	13	8	5	4	4
−50	4	4	7	16	W	W	W	W	13	7	5	4
−60	5	7	14	W	W	W	W	W	W	8	5	5
−70	6	10	W	W	W	W	W	W	W	7	5	5
−80	10	W	W	W	W	W	W	W	W	12	7	7
−90	W	W	W	W	W	W	W	W	W	W	W	15

**Table 2:** Daily exposure times in minutes, mean for the latitude given, for maintenance of 25OHD levels under climatological clear-sky conditions, clear horizon, at noon. Skin type V, 35% skin area exposed, vertical body, averaged over all sides exposed simultaneously. W = Vitamin D Winter.

Lat/Month	Jan	Feb	Mar	Apr	May	Jun	Jul	Aug	Sep	Oct	Nov	Dec
90	W	W	W	W	W	W	W	W	W	W	W	W
80	W	W	W	W	W	34	36	W	W	W	W	W
70	W	W	W	W	28	23	23	32	W	W	W	W
60	W	W	W	30	20	16	16	20	35	W	W	W
50	W	W	31	19	15	13	12	14	20	38	W	W
40	W	28	18	13	11	11	10	10	13	20	36	W
30	20	14	11	10	10	10	10	9	10	12	18	23
20	11	9	8	9	11	11	11	10	9	9	11	13
10	8	7	8	11	9	9	9	10	9	8	8	9
0	7	7	9	8	8	8	8	8	10	8	7	7
−10	7	8	8	8	9	10	10	8	9	10	8	8
−20	9	8	8	9	12	15	14	11	9	9	10	10
−30	8	8	9	13	20	26	25	18	13	10	9	9
−40	8	9	12	20	38	W	W	32	19	13	10	9
−50	10	11	18	40	W	W	W	W	32	18	13	10
−60	13	18	34	W	W	W	W	W	46	21	14	12
−70	16	26	W	W	W	W	W	W	W	18	12	12
−80	25	W	W	W	W	W	W	W	W	31	17	18
−90	W	W	W	W	W	W	W	W	W	W	W	37

**Table 3:** Daily exposure times in minutes, mean for the latitude given, for maintenance of 25OHD levels under climatological all-sky conditions, clear horizon, at noon. White skin (ST I–IV), 35% skin area exposed, vertical body, averaged over all sides exposed simultaneously. W = Vitamin D Winter. ND = no cloud data available.

Lat/Month	Jan	Feb	Mar	Apr	May	Jun	Jul	Aug	Sep	Oct	Nov	Dec
90	ND	ND	ND	ND	ND	ND	ND	ND	ND	ND	ND	ND
80	ND	ND	ND	ND	ND	ND	ND	ND	ND	ND	ND	ND
70	W	W	W	W	18	15	13	21	W	W	W	W
60	W	W	W	16	11	8	8	12	20	W	W	W
50	W	W	17	10	8	6	6	7	10	20	W	W
40	W	14	9	7	5	5	4	5	6	10	18	W
30	9	6	5	4	4	4	4	4	4	5	8	11
20	5	4	3	4	5	4	5	4	4	4	5	6
10	3	3	3	5	4	4	4	5	4	4	4	4
0	3	3	5	4	4	4	4	4	5	4	3	3
−10	4	4	4	4	4	4	4	4	4	5	4	4
−20	4	4	3	4	5	6	6	5	4	4	5	5
−30	4	3	4	6	9	12	11	8	6	5	4	4
−40	4	4	6	11	21	W	W	17	10	7	5	5
−50	6	7	11	24	W	W	W	W	19	11	7	6
−60	8	11	22	W	W	W	W	W	20	10	6	7
−70	ND	ND	W	W	W	W	W	W	W	9	5	6
−80	ND	ND	ND	ND	ND	ND	ND	ND	ND	ND	ND	ND
−90	ND	ND	ND	ND	ND	ND	ND	ND	ND	ND	ND	ND

**Table 4:** Daily exposure times in minutes, mean for the latitude given, for maintenance of 25OHD levels under climatological all-sky conditions, clear horizon, at noon. Skin type VI, 35% skin area exposed, vertical body, averaged over all sides exposed simultaneously. W = Vitamin D Winter. ND = no cloud data available.

Lat/Month	Jan	Feb	Mar	Apr	May	Jun	Jul	Aug	Sep	Oct	Nov	Dec
90	ND	ND	ND	ND	ND	ND	ND	ND	ND	ND	ND	ND
80	ND	ND	ND	ND	ND	ND	ND	ND	ND	ND	ND	ND
70	W	W	W	W	71	61	54	83	W	W	W	W
60	W	W	W	64	44	32	34	48	82	W	W	W
50	W	W	67	41	30	25	25	28	41	78	W	W
40	W	55	37	27	21	19	18	18	23	39	72	W
30	37	25	19	17	17	18	17	15	16	21	33	43
20	19	16	13	15	18	18	19	17	15	16	19	22
10	14	12	13	18	16	16	17	20	17	15	15	15
0	13	14	19	16	15	15	15	15	18	15	14	14
−10	14	17	16	15	15	17	17	15	15	19	16	15
−20	17	15	14	16	20	26	24	19	16	17	19	18
−30	15	14	16	23	36	50	44	33	23	19	18	18
−40	16	18	25	43	84	W	W	69	41	27	21	19
−50	24	28	44	97	W	W	W	W	77	43	29	24
−60	31	45	87	W	W	W	W	W	79	38	24	27
−70	ND	ND	W	W	W	W	W	W	W	35	21	25
−80	ND	ND	ND	ND	ND	ND	ND	ND	ND	ND	ND	ND
−90	ND	ND	ND	ND	ND	ND	ND	ND	ND	ND	ND	ND

## Data Availability

The underlying global UV climatology on which this work is based are available from https://www.temis.nl/uvradiation/UVarchive.php. The equations used subsequently, and the data on which they are based, are available in the references provided below. The set of tables of exposure times generated by this work is available in the text and Supplementary Materials.

## Supplementary Materials

The following supporting information can be downloaded at: https://www.mdpi.com/article/10.3390/nu16101489/s1, Supplementary material consists of final maintenance dose tables (vertical body, 35% skin area exposed) not included in the main text (ST VI for clear-sky and ST V for all-sky conditions): Tables S1 and S2. We also show the intermediate step of horizontal body maintenance dose times, two sides (front and back), for clear-sky conditions (Tables S3–S5) and all-sky conditions (Tables S6–S8). Figures illustrate the range of maintenance doses for the month of March for Europe, Asia, Australia, and South America (Figures S1–S4).
