# Supplementary material for: Globally Estimated UVB Exposure Times Required to Maintain Sufficiency in Vitamin D Levels
Source: Nutrients. 2024 May 15;16(10):1489. doi: 10.3390/nu16101489 (PMC11124381; doi:10.3390/nu16101489)
Supplement: Supplementary file 1 [file nutrients-16-01489-s001.zip › nutrients-2997489-supplementary.pdf]

Globally Estimated UVB Exposure Times Required to Maintain Sufficiency in Vitamin D Levels

Richard C. Kift and Ann R. Webb *

Department of Earth and Environmental Sciences, University of Manchester, Manchester M13 9PL, UK; richard.kift@manchester.ac.uk
* Correspondence: ann.webb@manchester.ac.uk; Tel.: +44-161-306-3917

Supplementary Material.

The supplementary material covers additional tables of daily maintenance exposure time, covering the vertical exposure times not in the main text (Tables S1 and S2), and the horizontal exposure times for all skin types in both clear sky (Tables S3 to S5) and all sky (Tables S6-S8) conditions.

Additional figures show March maintenance dose exposure time maps for regions of the world not represented in the main text: Europe (Figure S1), South Asia (Figure S2), Australia (Figure S3), and South America (Figure S4). Note the colour scales differ for the different regions of the world.

Table S1. Daily exposure times in minutes, mean for the latitude given, for maintenance of 25OHD levels under climatological clear-sky conditions, clear horizon, at noon. Skin type VI, 35% skin area exposed, vertical body, averaged over all sides exposed simultaneously. W = Vitamin D Winter.

Lat/ Mth	Jan	Feb	Mar	Apr	May	Jun	Jul	Aug	Sep	Oct	Nov	Dec
90	W	W	W	W	W	W	W	W	W	W	W	W
80	W	W	W	W	W	55	58	W	W	W	W	W
70	W	W	W	W	45	36	37	52	W	W	W	W
60	W	W	W	47	32	26	26	32	57	W	W	W
50	W	W	49	31	23	20	19	22	32	61	W	W
40	W	45	29	21	18	17	16	17	21	31	58	W
30	32	23	17	15	16	17	16	14	15	20	29	38
20	18	15	13	15	18	17	18	16	14	15	18	21
10	13	11	12	17	14	14	14	16	15	13	13	14
0	11	12	15	13	13	13	13	13	16	13	12	11
-10	12	13	13	13	14	16	15	13	14	16	13	12
-20	14	13	12	14	19	23	23	18	15	15	16	15
-30	13	12	14	20	31	42	40	29	20	16	15	15
-40	13	14	19	32	61	W	W	52	31	21	16	14
-50	15	18	30	64	W	W	W	W	51	29	20	16
-60	21	29	55	W	W	W	W	W	73	33	22	19
-70	25	41	W	W	W	W	W	W	W	29	19	20
-80	41	W	W	W	W	W	W	W	W	49	27	29
-90	W	W	W	W	W	W	W	W	W	W	W	59

Table S2. Daily exposure times in minutes, mean for the latitude given, for maintenance of 25OHD levels under climatological all-sky conditions, clear horizon at noon. Skin type V, 35% skin area exposed, vertical body, averaged over all sides exposed simultaneously. W = Vitamin D Winter. ND = no cloud data available.

Lat/ Mth	Jan	Feb	Mar	Apr	May	Jun	Jul	Au g	Sep	Oct	No v	Dec
90	ND	ND	ND	ND	ND	ND	ND	ND	ND	ND	ND	ND
80	ND	ND	ND	ND	ND	ND	ND	ND	ND	ND	ND	ND
70	W	W	W	W	44	38	34	52	W	W	W	W
60	W	W	W	40	28	20	21	30	51	W	W	W
50	W	W	42	26	19	15	16	18	25	49	W	W
40	W	34	23	17	13	12	11	11	15	25	45	W
30	23	16	12	11	11	11	10	10	10	13	21	27
20	12	10	8	9	11	11	12	10	9	10	12	14
10	8	7	8	11	10	10	11	12	11	9	9	9
0	8	8	12	10	10	9	10	9	11	10	8	8
-10	9	10	10	9	10	11	10	9	10	12	10	10
-20	11	10	9	10	13	16	15	12	10	10	12	11
-30	9	9	10	14	23	31	28	21	14	12	11	11
-40	10	11	16	27	52	W	W	43	26	17	13	12
-50	15	17	28	60	W	W	W	W	48	27	18	15
-60	20	28	54	W	W	W	W	W	49	24	15	17
-70	ND	ND	W	W	W	W	W	W	W	22	13	15
-80	ND	ND	ND	ND	ND	ND	ND	ND	ND	ND	ND	ND
-90	ND	ND	ND	ND	ND	ND	ND	ND	ND	ND	ND	ND

Table S3. Daily exposure times in minutes, mean for the latitude given, for maintenance of 25OHD levels under climatological clear-sky conditions, clear horizon, at noon. Skin type I-IV, 35% skin area exposed, horizontal body, 2 sides exposed. W = Vitamin D Winter.

Lat/ Mth	Jan	Feb	Mar	Apr	May	Jun	Jul	Aug	Sep	Oct	Nov	Dec
90	W	W	W	W	W	W	W	W	W	W	W	W
80	W	W	W	W	W	6	6	W	W	W	W	W
70	W	W	W	W	5	4	4	6	W	W	W	W
60	W	W	W	5	3	3	3	3	6	W	W	W
50	W	W	5	3	2	2	2	2	3	7	W	W
40	W	5	3	2	2	1	1	2	2	3	6	W
30	3	2	2	1	1	1	1	1	2	2	3	4
20	2	2	1	1	1	1	1	1	1	1	2	2
10	1	1	1	1	1	1	1	1	1	1	1	1
0	1	1	1	1	1	1	1	1	1	1	1	1
-10	1	1	1	1	1	2	2	1	1	1	1	1
-20	1	1	1	1	2	2	2	2	1	1	1	1
-30	1	1	1	2	3	5	4	3	2	2	1	1
-40	1	1	2	3	7	W	W	6	3	2	2	1
-50	2	2	3	7	W	W	W	W	6	3	2	2
-60	2	3	6	W	W	W	W	W	8	4	2	2
-70	3	5	W	W	W	W	W	W	W	3	2	2
-80	5	W	W	W	W	W	W	W	W	6	3	3
-90	W	W	W	W	W	W	W	W	W	W	W	7

Table S4. Daily exposure times in minutes, mean for the latitude given, for maintenance of 25OHD levels under climatological clear-sky conditions, clear horizon, at noon. Skin type V, 35% skin area exposed, horizontal body, 2 sides exposed. W = Vitamin D Winter.

Lat/Mth	Jan	Feb	Mar	Apr	May	Jun	Jul	Aug	Sep	Oct	Nov	Dec
90	W	W	W	W	W	W	W	W	W	W	W	W
80	W	W	W	W	W	15	16	W	W	W	W	W
70	W	W	W	W	12	10	10	14	W	W	W	W
60	W	W	W	13	8	7	7	9	16	W	W	W
50	W	W	13	8	6	5	5	6	9	17	W	W
40	W	12	8	5	4	4	4	4	5	8	16	W
30	9	6	4	4	3	3	3	3	4	5	8	10
20	5	4	3	3	3	3	3	3	3	4	5	6
10	3	3	3	3	3	3	3	3	3	3	3	4
0	3	2	2	3	3	3	3	3	3	3	3	3
-10	2	2	2	3	4	4	4	3	3	3	3	3
-20	2	2	3	4	5	6	6	5	4	3	3	3
-30	2	3	4	5	8	11	11	8	5	4	3	3
-40	3	3	5	9	17	W	W	14	8	5	4	3
-50	4	5	8	18	W	W	W	W	14	8	5	4
-60	6	8	15	W	W	W	W	W	21	9	6	5
-70	7	11	W	W	W	W	W	W	W	8	5	5
-80	11	W	W	W	W	W	W	W	W	15	8	8
-90	W	W	W	W	W	W	W	W	W	W	W	17

Table S5. Daily exposure times in minutes, mean for the latitude given, for maintenance of 25OHD levels under climatological clear-sky conditions, clear horizon, at noon. Skin type VI, 35% skin area exposed, horizontal body, 2 sides exposed. W = Vitamin D Winter.

Lat/Mth	Jan	Feb	Mar	Apr	May	Jun	Jul	Aug	Sep	Oct	Nov	Dec
90	W	W	W	W	W	W	W	W	W	W	W	W
80	W	W	W	W	W	24	26	W	W	W	W	W
70	W	W	W	W	20	15	16	23	W	W	W	W
60	W	W	W	20	13	11	11	14	25	W	W	W
50	W	W	21	13	9	8	8	9	14	27	W	W
40	W	19	12	9	7	6	6	7	9	14	26	W
30	14	10	7	6	5	5	5	5	6	8	13	16
20	8	6	5	5	4	5	5	4	5	6	8	9
10	5	4	4	4	4	5	5	4	4	5	5	6
0	4	4	4	4	5	5	5	4	4	4	4	4
-10	4	4	4	5	6	6	6	5	5	4	4	4
-20	4	4	4	6	8	10	10	7	6	5	4	4
-30	4	4	6	8	13	18	17	12	8	6	5	4
-40	5	5	8	14	27	W	W	23	13	8	6	5
-50	6	7	13	29	W	W	W	W	22	12	8	6
-60	9	12	24	W	W	W	W	W	33	14	9	8
-70	11	18	W	W	W	W	W	W	W	13	8	8
-80	18	W	W	W	W	W	W	W	W	23	12	13
-90	W	W	W	W	W	W	W	W	W	W	W	27

Table S6. Daily exposure times in minutes, mean for the latitude given, for maintenance of 25OHD levels under climatological all-sky conditions, clear horizon, at noon. White skin (ST I-IV), 35% skin area exposed, horizontal body, 2 sides exposed W = Vitamin D Winter. ND = no cloud data available.

Lat/ Mth	Jan	Feb	Mar	Apr	May	Jun	Jul	Aug	Sep	Oct	Nov	Dec
90	ND	ND	ND	ND	ND	ND	ND	ND	ND	ND	ND	ND
80	ND	ND	ND	ND	ND	ND	ND	ND	ND	ND	ND	ND
70	W	W	W	W	8	7	6	9	W	W	W	W
60	W	W	W	7	5	3	4	5	9	W	W	W
50	W	W	7	4	3	2	2	3	4	9	W	W
40	W	6	4	3	2	2	2	2	2	4	8	W
30	4	3	2	2	1	1	1	1	2	2	4	5
20	2	2	1	1	1	1	1	1	1	2	2	2
10	1	1	1	1	1	1	1	1	1	1	1	2
0	1	1	1	1	1	1	1	1	1	1	1	1
-10	1	1	1	1	2	2	2	1	1	1	1	1
-20	1	1	1	2	2	3	3	2	2	1	1	1
-30	1	1	2	2	4	5	5	3	2	2	1	1
-40	1	2	3	5	9	W	W	8	4	3	2	2
-50	2	3	5	11	W	W	W	W	8	5	3	2
-60	3	5	10	W	W	W	W	W	9	4	3	3
-70	ND	ND	W	W	W	W	W	W	W	4	2	3
-80	ND	ND	ND	ND	ND	ND	ND	ND	ND	ND	ND	ND
-90	ND	ND	ND	ND	ND	ND	ND	ND	ND	ND	ND	ND

Table S7. Daily exposure times in minutes, mean for the latitude given, for maintenance of 25OHD levels under climatological all-sky conditions, clear horizon, at noon. Skin Type V, 35% skin area exposed, horizontal body, 2 sides exposed. W = Vitamin D Winter, ND = no cloud data available.

Lat/ Mth	Jan	Feb	Mar	Apr	Ma y	Jun	Jul	Aug	Sep	Oct	No v	Dec
90	ND	ND	ND	ND	ND	ND	ND	ND	ND	ND	ND	ND
80	ND	ND	ND	ND	ND	ND	ND	ND	ND	ND	ND	ND
70	W	W	W	W	19	16	14	23	W	W	W	W
60	W	W	W	17	12	8	9	13	23	W	W	W
50	W	W	18	11	8	6	6	7	11	22	W	W
40	W	15	10	7	5	4	4	4	6	11	20	W
30	10	7	5	4	3	3	3	3	4	6	9	12
20	5	4	3	3	3	3	3	3	3	4	5	6
10	3	3	3	3	3	3	4	3	3	3	4	4
0	3	3	3	3	3	4	4	3	3	3	3	3
-10	3	3	3	3	4	4	4	4	3	3	3	3
-20	3	3	3	4	5	7	6	5	4	3	3	3
-30	3	3	4	6	10	14	12	9	6	4	4	3
-40	4	4	7	12	23	W	W	19	11	7	5	4
-50	6	7	12	27	W	W	W	W	21	11	7	6
-60	8	12	24	W	W	W	W	W	22	10	6	7
-70	ND	ND	W	W	W	W	W	W	W	10	6	7
-80	ND	ND	ND	ND	ND	ND	ND	ND	ND	ND	ND	ND
-90	ND	ND	ND	ND	ND	ND	ND	ND	ND	ND	ND	ND

Table S8. Daily exposure times in minutes, mean for the latitude given, for maintenance of 25OHD levels under climatological all-sky conditions, clear horizon, at noon. Black skin (ST VI), 35% skin area exposed, horizontal body, 2 sides exposed. W = Vitamin D Winter, ND = no cloud data available.

Lat/ Mth	Jan	Feb	Mar	Apr	Ma y	Jun	Jul	Aug	Sep	Oct	Nov	Dec
90	ND	ND	ND	ND	ND	ND	ND	ND	ND	ND	ND	ND
80	ND	ND	ND	ND	ND	ND	ND	ND	ND	ND	ND	ND
70	W	W	W	W	31	26	23	37	W	W	W	W
60	W	W	W	28	19	13	14	20	36	W	W	W
50	W	W	29	17	12	10	10	12	17	35	W	W
40	W	24	15	11	8	7	7	7	10	17	32	W
30	16	11	8	6	6	5	5	5	6	9	14	19
20	8	6	5	5	5	5	5	5	5	6	8	9
10	5	5	4	4	5	5	6	5	5	5	6	6
0	5	5	5	5	6	6	6	5	5	5	5	5
-10	5	5	5	6	6	7	7	6	5	5	5	5
-20	4	5	5	6	8	11	10	8	6	5	5	5
-30	5	5	7	10	16	22	19	14	9	7	6	5
-40	6	7	10	19	37	W	W	30	17	11	8	7
-50	10	12	19	43	W	W	W	W	34	18	12	10
-60	13	19	39	W	W	W	W	W	36	17	10	11
-70	ND	ND	W	W	W	W	W	W	W	16	9	11
-80	ND	ND	ND	ND	ND	ND	ND	ND	ND	ND	ND	ND
-90	ND	ND	ND	ND	ND	ND	ND	ND	ND	ND	ND	ND

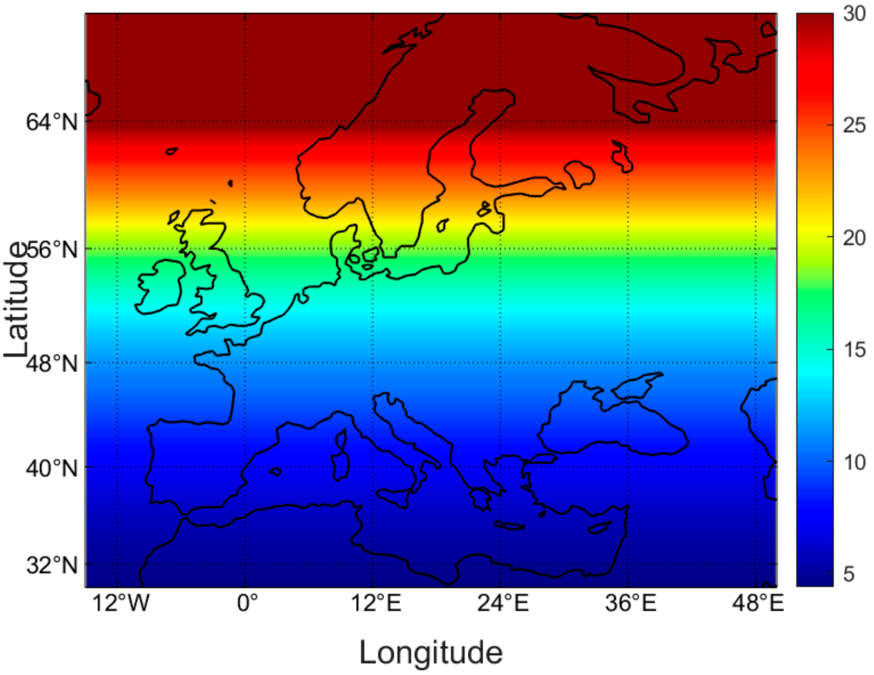

Figure S1. March maintenance dose times (mins) for clear sky conditions at noon, white skin types, 35% skin area exposed, vertical body, average all sides, for Europe. Note the different colour scales. The high latitude brown region indicates where the UVI is below the defined VDW values.

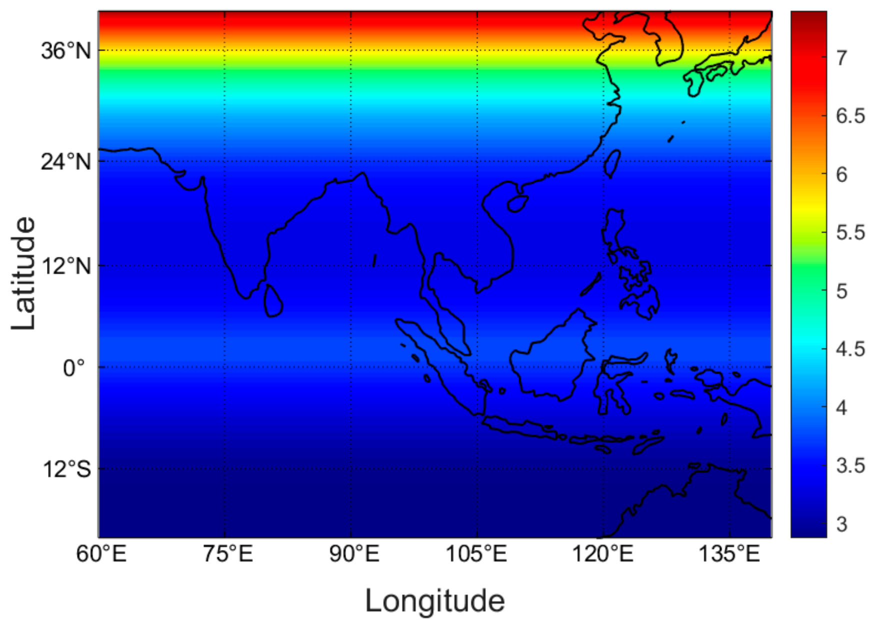

Figure S2. March maintenance dose times (mins) for clear sky conditions at noon, white skin types, 35% skin area exposed, vertical body, average all sides, for Southern Asia. Note the different colour scales.

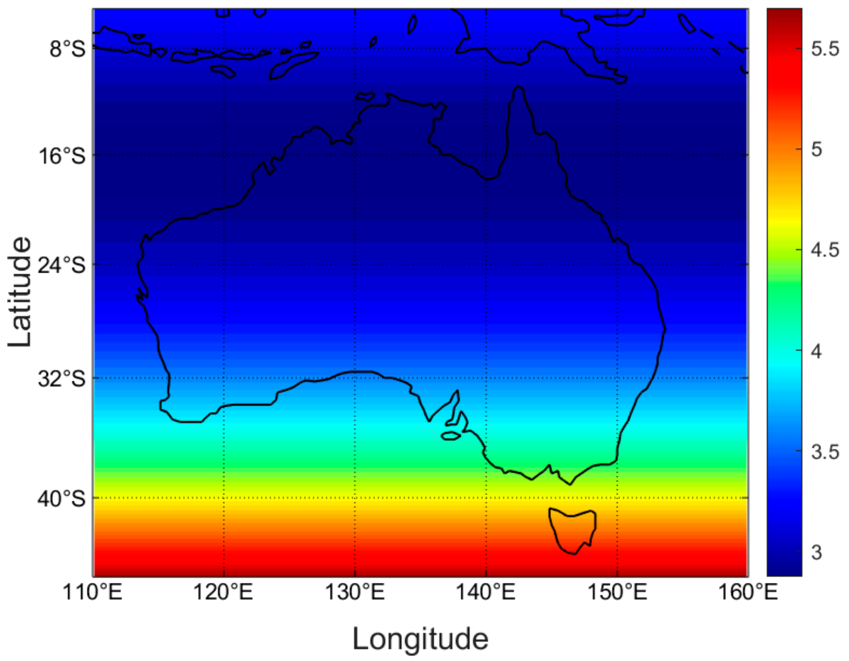

Figure S3. March maintenance dose times (mins) for clear sky conditions at noon, white skin types, 35% skin area exposed, vertical body, average all sides, for Australia. Note the different colour scales.

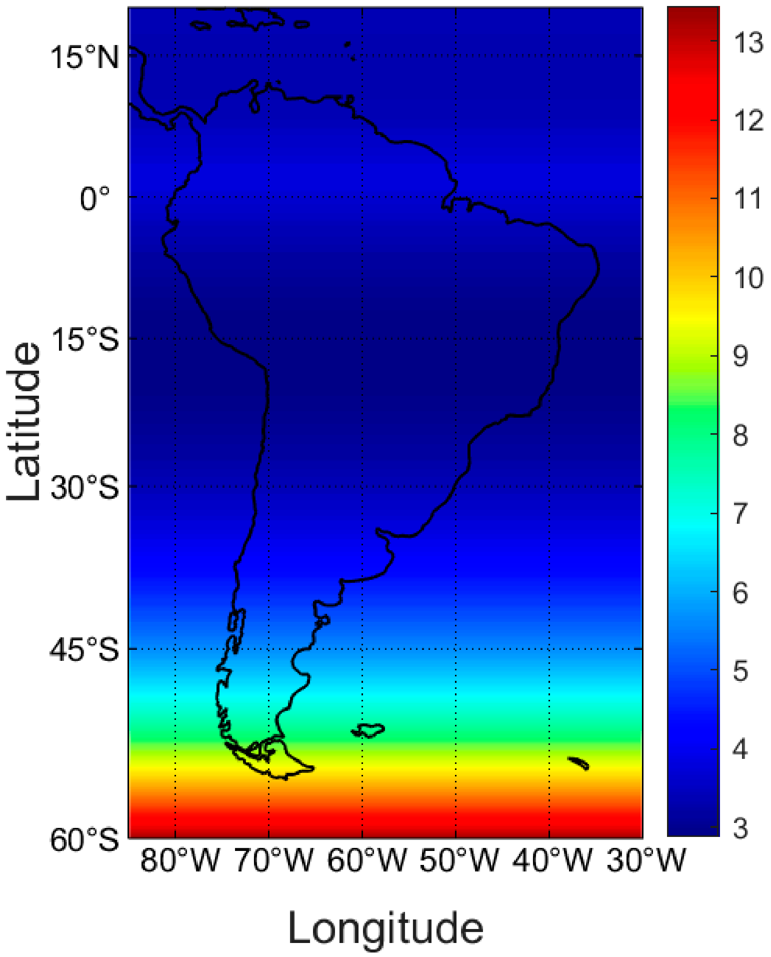

Figure S4. March maintenance dose times (mins) for clear sky conditions at noon, white skin types, 35% skin area exposed, vertical body, average all sides, for South America. Note the different colour scales.
